# Prevalence and antimicrobial susceptibility pattern of methicillin-resistant *Staphylococcus aureus* in Assam

**DOI:** 10.4103/0972-5229.58542

**Published:** 2009

**Authors:** Lahari Saikia, Reema Nath, Basabdatta Choudhury, Mili Sarkar

**Affiliations:** **From** Department of Microbiology, Assam Medical College, Dibrugarh-786 002, India

**Keywords:** Antibiotics, macrolids lincosamides type B streptogramins resistance, methicillin-resistant *Staphylococcus aureus*

## Abstract

**Aims:**

Methicillin-resistant *Staphylococcus aureus* (MRSA) has become a serious problem in intensive care units, because of development of multiresistance, and also intrinsic resistance to β-lactam antibiotics. The present study was carried out to investigate the prevalence of MRSA and their rate of resistance to different antistaphylococcal antibiotics.

**Materials and Methods:**

Between January 2007 and February 2008, the clinical specimens submitted at the microbiology laboratory were processed and all *S*. *aureus* isolates were included in this study. All isolates were identified morphologically and biochemically by standard laboratory procedures and antibiotic susceptibility pattern was determined by modified Kirby Bauer disc diffusion method.

**Results:**

Methicillin resistance was observed in 34.78% of isolates, of which 37.5% were found to be resistant to all commonly used antibiotics. In MRSA isolates, 50% had constitutive resistance, 9.38% had inducible MLS_B_ resistance and 18.75% had MS phenotype.

**Conclusions:**

There is a progressive increase in MRSA prevalence in the country but the present rate is still low in comparison to values found in some other institutes. The rate of inducible MLS_B_ resistance was also lower in comparison with findings from other parts of the country.

## Introduction

Methicillin-resistant *Staphylococcus aureus* (MRSA) is one of the most important nosocomial pathogens and has emerged as a serious threat to public health world wide.[1] Because of their multiresistance properties, with intrinsic resistance to all β-lactam antibiotics, there remain limited choices of antimicrobial agents to treat many serious life-threatening infections caused by MRSA, leading to prolonged stay of such patients in the ICU and hospital, and increased cost of care. The emergence of these resistant strains represents a consequential response to selective pressures imposed by antimicrobial chemotherapy and once established, they are difficult to control and eradicate. The knowledge of prevalence of MRSA and their antibiotic sensitivity pattern in any environment becomes necessary for selection of appropriate treatment for these patients. The aim of this study is to determine the prevalence and antimicrobial susceptibility pattern of MRSA in a tertiary care hospital in Assam.

## Materials and Methods

*Staphylococcus aureus* isolates from routine clinical specimens submitted at the microbiology laboratories from January 2007 to February 2008 were included in this study. All isolates were identified morphologically and biochemically by standard laboratory procedures.[2]

The antibiotic susceptibility pattern was determined by modified Kirby Bauer disc diffusion method against the following antibiotics: Oxacillin (1 μg), penicillin (10 U), Cephalexin (30 μg), gentamicin (10 μg), amikacin (30 μg), trimethoprim/sulfamethoxazole (1.25/23.75 μg), ciprofloxacillin (5 μg), erythromycin (15 μg) and clindamycin (2 μg). The entire surface of the Mueller-Hinton agar (MHA) plate with 2% NaCl was covered with inoculums of *S*. *aureus*, turbidity matching 0.5 McFarland standard, by a sterile cotton swab stick and the plate was air-dried before antibiotics discs were laid on the surface. To determining inducible macrolids Lincosamides type B streptogramins (MLS_B_) resistance (D-test) erythromycin and clindamycin discs were placed 15-18 mm apart. A truncated or blunted clindamycin zone of inhibition (D-shape) indicated inducible resistance. All discs were obtained from Difco supplied by Becton Dickinson India Pvt. Ltd. *S*. *aureus* ATCC 25923 as sensitive and ATCC 43300 as oxacillin-resistant strain were used for standard control. The plates were incubated at 35°C for 24 hours. The diameter of the zone of inhibition was compared according to Clinical and Laboratory Standards Institute guidelines (CLSI).[3]

## Results

A total of 276 *S*. *aureus* strains were isolated from various clinical specimens. Of 276 isolates, 96 (34.78%) were found to be methicillin-resistant. Maximum isolation of MRSA was from pus/wound swabs (46.67%) followed by sputum/throat swab (42.86%). Table 1 depicts the antibiotic susceptibility data for all the *S*. *aureus* isolates. None of the MRSA isolates was found to be sensitive to penicillin and Cephalexin, while 10.56% and 25% of methicillin-sensitive *S*. *aureus* (MSSA) were sensitive to these antibiotics, respectively. MSSA isolates also revealed higher susceptibility to trimethoprim/sulfamethoxazole (38.89% vs. 3.12%), gentamicin (27.22% vs. 12.5%), amikacin (61.1% vs. 21.88%), ciprofloxacillin (48.33% vs. 12.5%), erythromycin (75% vs. 18.75%) and clindamycin (90.56% vs. 43.75%) as compared with MRSA. In MRSA isolates, 50% had constitutive resistance (resistance to both erythromycin and clindamycin), 9.38% had the inducible MLS_B_ resistance (flattening of the clindamycin zone adjacent to the erythromycin disc) and 18.75% had the MS phenotype (resistance to erythromycin and sensitive to clindamycin). In MSSA, 5% and 3.3% isolates were found to have the constitutive and inducible MLS_B_ resistance phenotypes respectively, while 23.33% exhibited the MS phenotype.

**Table 1:** Antibiotic susceptibility pattern of 276 *Staphylococcus aureus* isolates from clinical specimens received at microbiology department, January 2007-February 2008

Antimicrobials	Percentage of isolates sensitive	
	
	MSSA, n = 180 (65.21%)	MRSA, n = 96 (34.78%)
Oxacillin	100	0
Penicillin	10.56	0
Cephalexin	25	0
Trimethoprim/sulfamethoxazole	38.89	3.12
Gentamicin	27.22	12.5
Amikacin	61.1	21.88
Ciprofloxacillin	48.33	12.5
Erythromycin	75	18.75
Clindamycin	90.56	43.75

## Discussion

MRSA is a global phenomenon with a prevalence rate ranging from 2% in the Netherlands and Switzerland, to 70% in Japan and Hong Kong.[4,5] In this study, the prevalence of MRSA was found to be 34.78%, and this is higher than previous rates (23.6%) reported from the same institute.[6] A comparable prevalence rate of 31% and 38.56% were also reported from Tamil Nadu and Delhi, whereas in some studies the rate is comparatively low (19.56% in Nagpur) and in another study it was very high (80.89% in Indore).[7–10] Analysis from previous studies revealed a relationship between methicillin resistance and resistance to other antibiotics.[6,11] This study showed that all MRSA isolates were significantly less sensitive to antibiotics as compared with MSSA isolates. Many of the isolates (37.5%) were resistant to all antibiotics used. Anupurba *et al*. also observed that 32% of MRSA isolates are resistant to all commonly used antistaphylococcal agents except vancomycin.[12] Because of the resistance of MRSA to all commonly used antibiotics, it is necessary to test newer group of antibiotics such as vancomycin and teicoplanin routinely. Resistance to quinolones (ciprofloxacillin) was much higher (87.5%) in this study as compared with a previous study (22.8%) from the same institute.[6] The rapid emergence of ciprofloxacillin is probably due to the indiscriminate and empirical use of these drugs. Susceptibility to erythromycin and clindamycin were observed in 55.43% and 74.28%, respectively. MSSA isolates revealed higher susceptibility to erythromycin (75% vs. 18.75%) and clindamycin (90.56% vs. 43.75%) than MRSA. Both the constitutive (5% vs. 50%) and inducible resistance phenotypes (3.3% vs. 9.38%) were found to be significantly higher in MRSA isolates as compared with MSSA. A recent study in the All India Institute of Medical Science, New Delhi, observed that 10% of MSSA and 30% MRSA are D-test positive.[13] In our study, positive D-test was observed in 3.33% of MSSA and 9.38% in MRSA. A recent survey in South Africa observed inducible MLS_B_ phenotype in 10.8% of MSSA and 82% of MRSA, whereas constitutive MLS_B_ phenotype was identified in 1.4% of MSSA isolates but absent in all the MRSA.[14]

## Conclusions

These observations indicate that the incidence of constitutive and inducible MLS_B_ resistance in staphylococcal isolates varies by geographic region. The clinical microbiology laboratories should consider routine testing and reporting of inducible clindamycin resistance in *S*. *aureus* to prevent the possibility of clindamycin treatment failure. Because of the ability of these pathogens to acquire resistance to new classes of antimicrobial agents, surveillance on the antimicrobial susceptibility patterns is of utmost importance in understanding new and emerging resistance trends.
